# Atrial Fibrillation in Obstructive Sleep Apnea Patients: Mechanisms, Risk Factors, and Management Strategies

**DOI:** 10.7759/cureus.36282

**Published:** 2023-03-17

**Authors:** Rajagopal Sankaranarayanan, Arpit Bansal, Vishnu R Yanamaladoddi, Sai S Sarvepalli, Shree L Vemula, Saikumar Aramadaka, Raam Mannam

**Affiliations:** 1 Research, Narayana Medical College, Nellore, IND; 2 Research, A. C. Subba Reddy (ACSR) Government Medical College, Nellore, IND

**Keywords:** airway obstruction, sleep, osas, sleep apnea, continuous positive airway pressure (cpap), stroke, obstructive sleep apnea, atrial fibrillation

## Abstract

Obstructive sleep apnea (OSA) is identified by apnea or hypopnea of the upper respiratory tract, which is associated with decreased oxygen saturation or awakening from sleep. A severe and prevalent association with OSA is atrial fibrillation (AF). This review article outlined numerous studies to understand the pathogenic pathways linked with developing OSA-associated AF and the therapeutic and preventive options available to reduce AF. The article looked for multiple risk factors common to OSA and AF. In addition, it has reviewed several therapeutic modalities such as continuous positive air pressure (CPAP), weight loss, upper airway stimulation (UAS), and other novel treatment options to find their efficiency in decreasing the outcome of AF in OSA patients. Since OSA often goes undiagnosed, this article emphasizes the importance of early screening in patients with AF and other comorbid conditions such as obesity, advanced age, diabetes, hypertension, and many more. The article focuses on the importance of preventive approaches that can be easily implemented, such as behavioral modifications.

## Introduction and background

**Figure 1:**
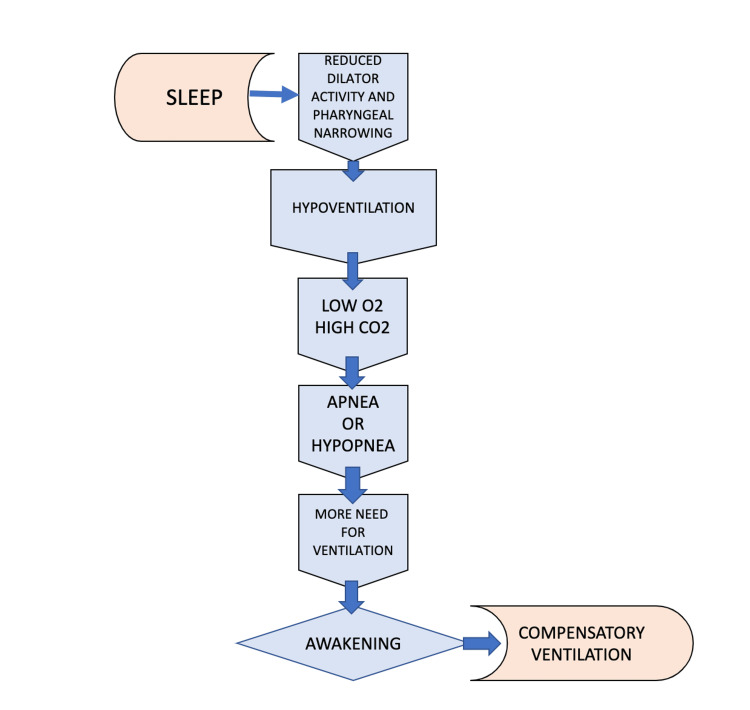
Obstructive sleep apnea pathogenesis Image credits - Dr. Rajagopal Sankaranarayanan

Obstructive sleep apnea (OSA) is identified by episodes of complete (apnea) or partial collapse (hypopnea) of the upper respiratory tract and is associated with decreased oxygen saturation or awakening from sleep [1]. By the definition of five or more events per hour, OSA affects one billion people worldwide [2]. In the United States, 25-30% of men and 9-17% of women are reported to meet the criteria for obstructive sleep apnea [3]. The prevalence also becomes more significant with age, and among individuals over 50, as many women as men come down with the disorder. The increased prevalence of OSA is linked with increased obesity rates varying from 14% to 55% [4]. OSA is found more in Hispanic, Black, and Asian communities [3]. Anatomical risk factors for OSA, such as obesity and upper airway soft tissue structure, indicate familial aggregation and a notable degree of heritability [5]. The tumor necrosis factor (TNF) polymorphism (TNFA rs1800629) was significantly associated with OSA under the allele frequency model [6]. Several risk factors are associated with OSA, including anatomic, non-anatomic, and additional factors. Pharyngeal constriction is propagated by anatomical factors, which include obesity, large neck circumference, bone, soft tissue, or vessels. Non-anatomic elements include advanced age, central fat distribution, supine sleeping position, and male gender, whereas alcohol use, smoking, and use of hypnotics and sedatives are some additional etiological components [7,8]. After falling asleep, there is reduced activity in the dilator muscles with loss of neuromuscular compensation resulting in narrow pharyngeal muscular activity and hypoventilation. A further decrease in upper airway muscle activity is followed by obstructive apnea or hypopnea. This follows hypoxia and hypercapnia, which leads to an increase in ventilator effort that causes awakening. Pharyngeal muscle activity and upper airway entry open rapidly upon arousal, and the patient hyperventilates to compensate for hypercapnia and hypoxia (Figure 1) [9]. 

**Figure 2:**
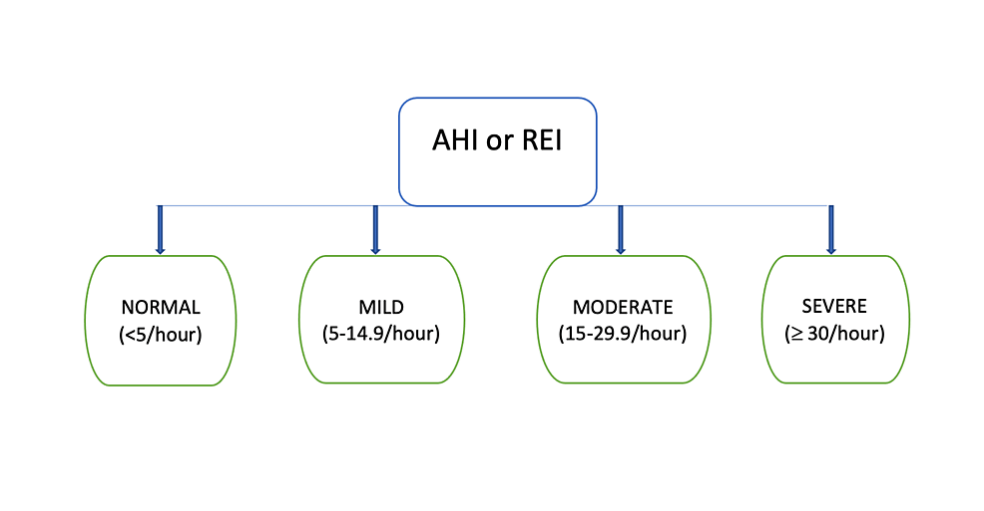
Obstructive sleep apnea severity determined by AHI or REI Image credits - Dr Rajagopal S. Narayanan AHI - apnea-hypopnea index, REI - respiratory event index

As a result, snoring, excessive daytime somnolence, choking or gasping at night, maintenance insomnia, night sweats, neurocognitive impairment, heartburn, morning headaches, and nocturia are considered symptoms particular to OSA [10]. The severity of OSA is decided by apnea-hypopnea index (AHI), or respiratory disturbance index (RDI) if polysomnography (PSG) is performed, or respiratory event index (REI) if an out-of-center sleeping test (OCST) is performed [11]. AHI or REI of <5/hour is considered normal; 5-14.9/hour is mild; 15-29.9/hour is moderate; and ≥ 30/hour is severe (Figure 2) [11].

There are a few screening tools, such as the Berlin questionnaire, the STOP-BANG questionnaire [12], and the preoperative questionnaire, to recognize patients at risk [11]. The Epworth Sleepiness Scale (ESS) is a subjective measure of sleepiness and is frequently used to screen for OSA [11].

PSG is the gold standard test to identify OSA, but when PSG is not achievable, OCST or home sleep apnea test (HSAT) are considered substitutes [11]. Positive airway pressure (PAP) is the optimal treatment for all degrees of OSA. PAP is primarily used as continuous PAP (CPAP). However, automated titration PAP (APAP) and bilevel PAP (BPAP) modes are also available for selected patients. Behavioral therapies include weight loss, position therapy, and avoiding alcohol and tranquilizers before bedtime [13]. Oral appliances (OA) or mandibular repositioning appliances (MRA) are designed to manage OSA by widening the upper airway by advancing the mandible forward [13]. The impact of OSA is not just limited to excessive daytime sleepiness and a rise in cerebrovascular or cardiovascular events. OSA also significantly affects other critical domains of quality of life like somatic sensation, physical function, emotional state, and social interaction, which aren't generally explored in the sleep laboratory. Thus it contributes significantly to obstacles in all aspects of what is often mentioned as " health-related quality of life" [14]. Sleep apnea can cause pulmonary hypertension, neurocognitive effects, inferior quality of life, car accidents, awakening headaches, childhood growth arrest, pregnancy-induced hypertension, fetal growth retardation, and poor sleep quality of bed partners [15].

Atrial fibrillation (AF) occurs in 5% of patients experiencing OSA [16]. OSA has also been recognized as a risk factor for the beginning and progression of AF and reduces the effectiveness of antiarrhythmic drugs, electric cardioversion (EC), and catheter ablation (CA) in AF [17]. There is increasing evidence that autonomic activation contributes to the pathogenesis of AF in OSA. Acute apnea episodes lead to sympathetic activation, shortening the duration of atrial load and promoting the onset of AF [17]. Data from clinical studies of patients with AF show CPAP and autonomic regulation for treating OSA-related AF [17,18]. This review article aims to target the clinical relationship between AF and OSA, explore the existing screening and upcoming management guidelines, and improve health goals and quality of life in such patients.

## Review

Mechanism

There are various theories regarding what might contribute to possible AF in patients with OSA.

Autonomic Dysfunction

First, autonomic dysfunction has been linked with the induction of AF in OSA patients. Repeated desaturation and reoxygenation activate carotid chemoreceptors and increase sympathetic basal tone. In addition, intermittent hypoxia helps activate atrial catecholaminergic channels. OSA patients have elevated plasma and urinary catecholamines, consistent with sympathetic activation. Dimsdale et al. in 1995 studied plasma and urinary catecholamines in 43 patients, including hypertensive and normotensive patients with and without sleep apnea with similar age and obesity levels. The study revealed that 24-hour urinary norepinephrine levels were significantly higher in sleep apnea patients (58.2 ng vs. 40.2 ng in non-apneic, p<0.002) and were notably increased during day and night. Plasma norepinephrine levels were not that significantly elevated in apneic patients compared to hypertensive patients during sleep and morning (p<0.05). Hence, these studies have suggested the amendment of the sympathetic nervous system during sleep apnea [19]. The role of vago-sympathetic innervation was also explored. A study was conducted by Ghias et al. to imitate sleep apnea-induced AF in an experimental model and to ascertain whether nerve ablation prevents AF. Thirty dogs anesthetized with sodium pentobarbital were ventilated with a positive pressure ventilator, and OSA was stimulated by turning off the ventilator for two minutes at the end of expiration. Ablation of the ganglionic plexus adjacent to the pulmonary veins (PV) of these dogs showed no detectable further induction of AF. They also exhibited the role of vagal sympathetic innervation in sleep apnea in AF and the critical role of the plexuses surrounding the PV [20].

Endothelial Dysfunction and Oxidative Stress

Another probable mechanism comprises the role of endothelial dysfunction and oxidative stress [21]. Patients with OSA experience recurrent hypoxemia with sympathetic activation and marked elevations in blood pressure, which can impair endothelial function. Kato et al. investigated eight patients with OSA (age 44±4 years) and nine obese control subjects (age 48±3 years), which established that patients with OSA have a weakening of resistance-vessel endothelium-dependent vasodilation [22]. A cross-sectional study by Yamauchi et al. was done to inspect the relationship between OSA severity and oxidative stress. Of the total of 128 participants, 70 participants had non-severe OSA (AHI ≤ 30), and 58 subjects had severe OSA (AHI ≥ 30). Urinary excretion of 8-hydroxy-2'-deoxyguanosine (8-OHdG) was quantified as an in vivo factor of oxidative stress. The results revealed that urinary 8-OHdG excretion was significantly elevated in the severe OSA group (p=0.03), concluding that OSA severity is independently allied with oxidative stress [21]. 

Oxidative stress and endothelial dysfunction are associated with AF development. This is thought to occur because of perturbation of ion channels, including slow inactivating sodium currents, K channel, and L-type calcium channel currents. These channels are involved in AF initiation and AF maintenance [23].

Intrathoracic Pressure

Intrathoracic pressure is another link between OSA and AF. The constriction and collapse of the upper airway cause repeated fluctuations in intrathoracic pressure with sustained respiratory efforts. The thin-walled atria may be the most at risk to these transmural forces, which over time may promote atrial enlargement, a risk factor for AF. These forces may also be principal elements in tissue stretching and remodeling at the PV ostium, a site believed to be the focal point of AF discharge spread [24].

Diastolic Dysfunction

The following studies connect diastolic dysfunction, AF, and OSA. Fung et al. studied 68 consecutive patients with PSG-confirmed OSA who underwent echocardiography. Left ventricular diastolic function was established by transparent valve pulse wave doppler echocardiography. The study demonstrated that diastolic dysfunction with abnormal relaxation patterns was shared in OSA patients. In this study, more severe sleep apnea is related to greater left ventricular diastolic dysfunction [25]. Tsang et al. conducted a study to determine whether diastolic dysfunction correlates with an increased risk of nonvalvular atrial fibrillation (NVAF) in older adults with no history of atrial arrhythmia. Of 840 patients (39% men; mean (±SD) age, 75 ± 7 years), 80 (9.5%) developed NVAF over a mean (±SD) follow-up of 4.1 ± 2.7 years. The inference was that the occurrence and severity of diastolic dysfunction was an independent predictor of newly verified NVAF in the elderly [26]. 

Inter-atrial Block and Atrial Remodelling

Interatrial block (IAB) (described as a P-wave duration of ≥120 m) is a well-known etiology of AF [27]. A study by Todd et al. demonstrated IAB was more prevalent in 144 patients with moderate-severe OSA (mean AHI = 56.2 ± 27.9) compared to a control group of 36 patients with mild or no OSA (mean AHI = 5.6 ± 3.6) with incidences of 34.7% vs. 0%, respectively; p<0.001. Electrical remodeling leading to IAB may be mediated mechanically by changes in intrathoracic pressure or sustained increases in sympathetic tone [27]. Long-term OSA was linked with elevated levels of circulating markers of inflammation, including C-reactive protein (CRP), intercellular adhesion molecule-1 (ICAM-1), interleukin-8 (IL-8), and monocyte chemoattractant protein-1 (MCP-1) [28-31]. Increased circulating inflammatory factors, such as CRP and interleukin-6 (IL-6), have been linked with greater risk for AF, postoperative AF occurrence after coronary artery bypass grafting, and AF recurrence after EC or CA [32] (Figure 2).

**Figure 3:**
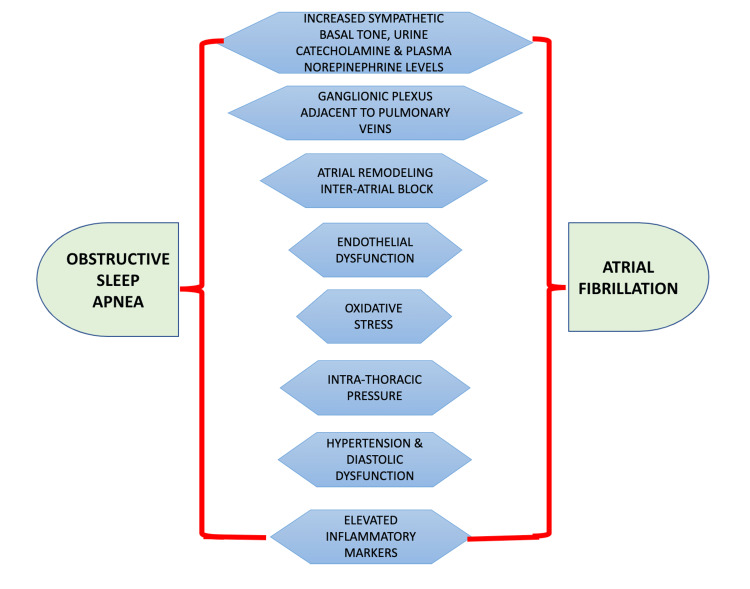
Summary of different mechanisms linking obstructive sleep apnea and atrial fibrillation Image credits - Dr Rajagopal S. Narayanan

Current evidence linking OSA and AF

A retrospective cohort study conducted by Gami et al. in 3542 Olmsted County adults without past or current AF mentioned for an initial diagnostic PSG from 1987 to 2003 were followed up for new-onset AF established by echocardiography for 4.7 years [33]. It aimed at recognizing whether obesity and OSA solitarily predict incident AF. Incident AF occurred in 133 subjects (cumulative probability 14%, 95% confidence interval (CI) 9% to 19%), and the study inferred obesity and magnitude of nocturnal oxygen desaturation in OSA are independent risk factors for incident AF in individuals <65 years of age [33].

OSA may independently exacerbate most of the risk factors that are part of the CHADSVASC scoring system (congestive heart failure (CHF), hypertension, age, diabetes, stroke, and vascular disease), which is a well-proven clinical tool for stroke risk assessment and used to decide upon treatment strategies of AF [34]. Wang et al. directed a cross-sectional study in China between January 2015 and October 2017. One thousand two hundred eighty-five elderly patients with OSA who undertook overnight PSG from multiple hospitals were enlisted for the study and were evaluated using 12-lead ECG or 24-h dynamic ECG. Their baseline demographics, clinical characteristics, sleep parameters, and medical history were determined. A total of 122 patients (9.5%), with 7.2% having paroxysmal AF and 2.3% with persistent AF, where the prevalence of AF was significantly greater with advanced age (p<0.05). Thus, laying out the evidence that AF is particularly frequent in older patients with OSA [35]. 

Peppard et al. conducted a prospective population-based study to acquire knowledge of the link between objectively measured sleep-disordered breathing (SDB) and hypertension [36]. Seven hundred nine participants from the Wisconsin Sleep Cohort Study were followed up for four years. They were assessed with 18-channel PSG as per AHI, and the odds ratio for the presence of hypertension was estimated. The odds ratio was 1.42 (95% (CI) 1.13 to 1.78) with AHI: 0.1-4.9; 2.03 (95% CI- 1.29 to 3.17) with AHI 5-14.9; 2.89 (95% CI -1.46 to 5.64) with AHI >15 [36]. Findings inferred that SDB is a probable cause of hypertension in the general population [36]. Hypertension leads to diastolic dysfunction, an independent predictor of NVAF, as mentioned in a study by Fung et al. [25]. Bradley et al. headed a study in 2003 including healthy human subjects and heart failure (HF) patients (seven of each) carrying out 15-second breath holds and obstructive apneas (OA) (Mueller maneuvers). In HF, simulated OA evoked more significant increases in sympathetic activity (p < 0.01) than holding breaths; hence the study offered a perception of the role of sleep apnea in the advancement of sympathetic activity and speeding up disease progression in HF [37].

Shahar et al. did a cross-sectional study between SDB and self-reported cardiovascular disease (CVD) in 6424 individuals who had overnight PSG at home. SDB was computed by AHI. Results demonstrated that SDB was connected strongly with stroke, with the relative odds (95% CI) of stroke being 1.58 (1.02-2.46) compared to other self-reported CVDs [38]. A study was published in 2006 by West et al. aiming to establish the prevalence of OSA in men with type 2 diabetes [39]. One thousand six hundred eighty-two men with type 2 diabetes from local hospitals and selected primary care practitioner databases were sent questionnaires regarding snoring, apneas, and daytime sleepiness under the Berlin questionnaire. Overnight oximetry was used to check for OSA, and comparisons were made [39]. A contrast of the oximetry results with men from a previous general population study (using only more than 10 >4% Sao (2) dips/hour to define OSA) demonstrated that the prevalence of OSA is notably higher in this diabetes population (17% v 6%, p<0.001). Thus, OSA is highly prevalent in type 2 diabetics [39]. Atherosclerosis is strongly associated with SDB. Schahab et al. studied 59 consecutive patients (mean age: 71.1 ± 9.8 years, 67.8 males) who were advanced peripheral artery disease (PAD) patients undergoing percutaneous revascularization [40]. Polygraphy was used to assess sleep apnea (SA) which revealed SA in 48 out of 59 (81.4%), of whom 60.4% showed obstructive-driven cause. Mean AHI and oxygen desaturation index (ODI) were 28.2 ± 19.5/h and 26.7 ± 18.8/h, respectively. Eighteen patients had AHI ≥ 30/h. For obstructive events, AHI correlated with PAD severity stages (p=0.042), inferring OSA correlation with severe PAD [40] (Figure 3).

**Figure 4:**
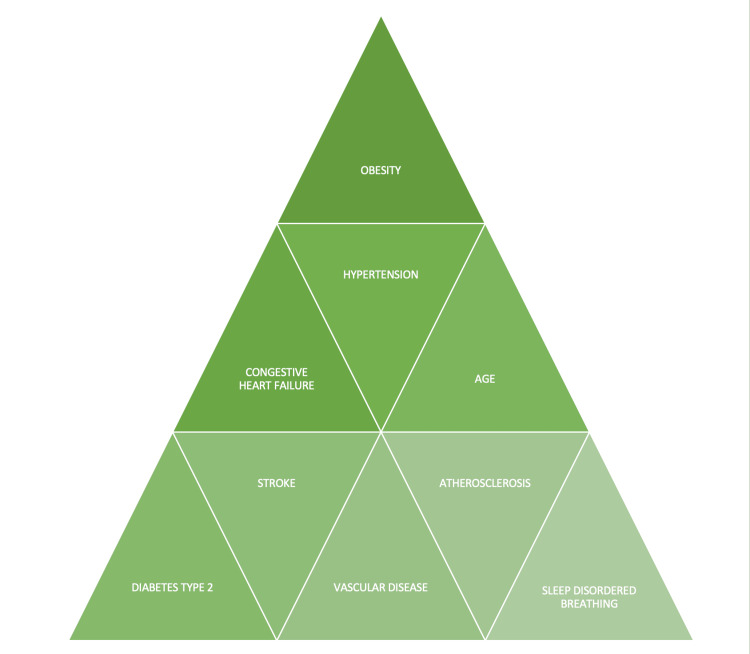
Risk factors common to obstructive sleep apnea and atrial fibrillation Image credits - Dr Rajagopal S. Narayanan

Management and treatment

OSA is a significant risk factor in the progression or recurrence of AF, and its treatment plays a role in the reversal of treatments available for AF. Moreover, the treatment of OSA is an essential part of AF management, especially when EC or pulmonary vein isolation (PVI) is used. The strength of the evidence justifies considering regular clinical screening for OSA before using rhythm control strategies [41]. OSA is a modifiable risk factor for preventing AF and is often underdiagnosed in patients with AF. A prospective study by Shapira-Daniels et al. included 188 patients with AF without a prior diagnosis of sleep apnea who were scheduled for AF ablation. The participants underwent home sleep apnea (HSA) testing and STOP-BANG questionnaires and were followed up for more than two years to estimate the impact of therapy [42]. One hundred fifty-five of 188 patients had a positive HSA test, of which 127 had a predominant obstructive component and 28 had mixed sleep apnea with a 15.2 ± 7.4% central component. The sensitivity and specificity of a STOP-BANG questionnaire were 81.2% and 42.4%, respectively. Hence the screening resulted in a high rate of long-term CPAP adherence [42]. Monahan et al., in 2012, studied the influence of OSA on the efficacy of anti-arrhythmic drug therapy (AAD) in patients with OSA and AF. Sixty-one patients (mean age 62 ± 15 years, 21 women) treated with AADs for symptomatic AF who underwent overnight PSG were studied. In conclusion, patients with severe OSA were less likely to respond to AAD therapy for AF than those with milder forms of OSA [43].

CPAP therapy

CPAP therapy is the most sought and mainstay therapy to treat OSA of all the treatment modalities available currently. OSA is crucial in the progression or recurrence of AF. A meta-analysis by Li et al. Analyzed in 2021 had nine studies with 14,812 patients. This investigation was an analysis of studies searched in the Cochrane Library, PubMed, EMBASE, EBSCO, OVID, and Web of Science databases till July 2020 to evaluate the recurrence or progression of AF in CPAP users, CPAP non-users, and patients without OSA. Findings revealed that CPAP therapy might reduce AF in patients not performing RA or direct cardioversion (DC) [44]. Another similar meta-analysis by Shukla et al. aimed to evaluate the cumulative effect of treatment of OSA with CPAP on AF recurrence and contrast between CPAP users and non-users with OSA. Also, it assessed the AF recurrence in CPAP users and non-users after PVI. Seven prospective cohort studies of 1,087 patients met the inclusion criteria. The study concluded that using CPAP is related to a significant reduction in AF recurrence in patients with OSA and is also consistent across patient populations, irrespective of whether they underwent PVI [45].

CPAP is a vital therapy in OSA patients undergoing PVI that protects against arrhythmias. Fein et al. studied 426 patients who underwent PVI between 2007 and 2010, among whom 62 patients had PSG-diagnosed OSA, where 32 patients were CPAP users, whereas the remaining were non-CPAP users. The study's main objective was to the impact of CPAP therapy on PVI outcomes in patients with OSA. Findings revealed that PVI offers limited value to OSA patients not treated by CPAP [46]. Another similar study by Li et al. in 2014 performed an online search and identified five studies involving 3743 patients with AF. Findings inferred that OSA was associated with AF recurrence even after catheter ablation (CA). Also, the efficacy of CA for AF was similar in patients without OSA and patients with OSA undergoing CPAP [47].

OSA is linked with atrial electric remodeling resulting in AF progression. Baranchuk et al., in 2013, at Kingston General Hospital, studied 19 severe OSA patients and 10 controls who came to the sleep disorder clinic to determine whether CPAP induces reverse atrial remodeling in patients with severe OSA with full PSG already done. The results showed a significant reverse in atrial electrical remodeling in patients with severe OSA treated with CPAP, represented by a substantial reduction in the average signal-wave duration (SAPW) [48]. Muscle sympathetic nerve activity (MSNA) is often associated with OSA, causing hypertension and cardiovascular morbidity [49]. Lundblad et al. Performed recordings of MSNA and blood oxygen level-dependent (BOLD) signal intensity of the brainstem, by high-resolution magnetic resonance imaging, in 15 controls and 13 subjects with OSA, before and after six months of CPAP treatment. CPAP treatment for six months reduced MSNA in subjects with OSA, proving effective [49]. CPAP may lower established risk factors for cardiovascular disease. Kohler et al. studied 102 males with moderate to severe OSA randomized to therapeutic (n=51) or sub-therapeutic (n= 51) CPAP treatment for four weeks to investigate its effects on 24-hour urinary catecholamine excretion, baroreflex sensitivity (BRS), arterial stiffness (augmentation index) and 24-hour ambulatory blood pressure (ABP). Results demonstrated that treatment of OSA with CPAP reduced sympathetic nerve activity, ABP, and arterial stiffness and increased arterial baroreflex sensitivity [50].

Weight reduction

Obesity, OSA, and AF are all interlinked entities and coexist in many scenarios. Pathak et al. Conducted a five-year follow-up cohort study on 1,415 consecutive patients with AF, where 825 had Body mass index (BMI) ≥27 kg/m2 and were offered weight management. After exclusion criteria screening, 355 were included in this analysis. The impact was determined on an AF severity scale and 7-day ambulatory monitoring. Results inferred that long-term sustained weight loss is associated with a significant decrease in AF burden and maintenance of sinus rhythm [51]. Abed et al. conducted a similar study in a partially blinded randomized control study between June 2010- December 2011 in Adelaide, Australia, where 150 patients were randomized and underwent a 15-month follow-up. Findings revealed a significant reduction in AF burden and severity and benefitted cardiac remodeling [52].

Upper airway stimulation

Upper airway stimulation (UAS) is also a therapy who have difficulty adhering to CPAP. In a prospective cohort design by Strollo Jr et al., the UAS device was surgically implanted [53]. The study included 126 participants. Primary outcome measures were done by AHI and ODI, whereas the secondary outcome measures were ESS, the Functional outcome of sleep questionnaire (FOSQ) [53]. Findings showed significant improvement in objective and subjective measurements of the severity of sleep apnea. UAS can be beneficial in treating OSA and AF in the future. However, further research is required [53]. The median AHI score at 12 months decreased by 68%, from 29.3 events per hour to 9.0 events per hour (P<0.001); the ODI score decreased by 70%, from 25.4 events per hour to 7.4 events per hour (P<0.001). Secondary outcome measures showed a reduction in the effects of sleep apnea and improved quality of life [53]. Two participants had a severe device-related adverse event and required repositioning and fixation of the neurostimulator to resolve the discomfort. A total of 33 serious adverse events were reported unrelated to the implantation procedure or implanted devices. Most nonserious adverse events related to the design (88%) occurred within 30 days after implantation. They were expected post-surgical events, including sore throat from intubation, pain at the incision site, and muscle soreness [53]. Overall the cohort had a reduction in the severity of obstructive sleep apnea, and the adverse-event profile was acceptable [53].

Novel therapeutic modalities

Renal denervation (RDN) was studied by Linz et al. in 2012 since OSA is associated with sympathovagal imbalance, AF, and post-apneic BP rises, and the investigation was done in 20 anesthetized pigs. Findings revealed RDN had anti-arrhythmic effects by decreasing negative tracheal pressure (NTP)-induced shortening of atrial effective refractory period (AERP) shortening and inhibits post-apneic BP rises associated with obstructive events [54]. Low-level vago-sympathetic trunk stimulation (LLVS) was studied by Gao et al. in 2015. Eleven rabbits received a tracheostomy under general anesthesia. The endotracheal tube was clamped to simulate OSA, and OSA was delivered every six minutes over four hours. Effective refractory period (ERP), blood pressure, intra-esophageal pressure, and blood gases (O2, CO2, pH) were measured before and after each episode of OSA. Results suggested the capability of LLVS to suppress ERP shortening and AF induced by OSA [55]. Low-level baroreceptor stimulation (LL-BRS) was studied by Linz et al. in 2016 on eight pigs who underwent tracheostomy under general anesthesia and received LL-BRS (at 80% of that slowing sinus rate) for three hours. Changes in AERP and AF-inducibility were verified during the applied NTP for two minutes before and at the end of the three-hour stimulation protocol. To conclude, LL-BRS suppressed NTP-induced AERP shortening and AF inducibility [56] (Table 1).

**Table 1:** Summary of the studies showing outcomes of various treatment modalities CPAP - continuous positive airway pressure; AF - atrial fibrillation; RA - radiofrequency ablation; DC - direct cardioversion; OSA - obstructive sleep apnea; PVI - pulmonary vein isolation; PSG - polysomnography; CA - catheter ablation; MSNA - muscle sympathetic nerve activity; BRS - baroreflex sensitivity; ABP - ambulatory blood pressure; BMI - body mass index; UAS - upper airway stimulation; RDN - renal denervation; LLVS - low-level vago-sympathetic nerve stimulation; BP - blood pressure; NTP - negative thoracic pressure; AERP - atrial effective refractory period; ERP - effective refractory period; LL-RBS - low-level baroreceptor stimulation

Reference		Design	Number of participants	Population	Conclusion
CPAP therapy					
Li et al. [44] (2021)		Meta-analysis	14812	Analysis of nine studies from several databases till July 2020, in CPAP users, CPAP non-users, patients without OSA	CPAP might reduce AF in patients irrespective of whether they perform RA or DC
Shukla et al. [45] (2015)		Meta-analysis	1087	Analysis of seven prospective cohort studies, to assess the cumulative effect of CPAP on AF recurrence in patients with OSA	CPAP causes a significant reduction of AF recurrence in patients with OSA, irrespective of whether they underwent PVI
Fein et al. [46] (2013)		-	426	patients who underwent PVI between 2007 and 2010; 62-PSG diagnosed OSA; 32 CPAP users, 30 CPAP non-users. To study the impact of CPAP on PVI outcomes in OSA patients	PVI offers limited value to OSA patients not treated by CPAP
Li et al. [47] (2014)		Online search study	3743	identified five studies with AF patients and studied impact of CPAP on patients undergoing RA with OSA	Efficacy of CA for AF was similar in patients without OSA and patients with OSA who underwent CPAP
Baranchuk et al. [48] (2013)		Hospital-based study	29	19 severe OSA patients, 10 controls at Kingston General Hospital, to determine whether CPAP induced reverse atrial remodeling in patients with severe OSA	Significant reversal in atrial electrical remodeling in patients with severe OSA and substantial reduction in SAPW
Lundblad et al. [49] (2015)		-	28	15 controls and 13 subjects with OSA, to study effect of CPAP on MSNA in patients with OSA; before and after 6 months of CPAP treatment	CPAP treatment for six months reduced MSNA in subjects with OSA
Kohler et al. [50] (2008)		-	102	male population with moderate to severe OSA; therapeutic (n=51), sub-therapeutic (n=51), CPAP treatment for 4 weeks to investigate effects on 24-hour catecholamine secretion, BRS, 24-hour ABP, arterial stiffness (augmentation index)	CPAP treatment reduced sympathetic nerve activity, ABP, arterial stiffness and increased arterial baroreflex sensitivity
Weight reduction					
Pathak et al. [51] (2015)		Prospective cohort study	1415	Of 1415 consecutive patients with AF, 825 had BMI >27 kg meter sq. After exclusion criteria, 355 were included in analysis and offered weight management	Long term sustained weight loss showed a significant decrease in AF burden and maintenance of sinus rhythm
Abed et al. [52] (2013)		Randomized control trial	150	partially blinded trial was conducted between June 2010 -December 2011 in Adelaide, Australia with 150 patients with 15 month follow up and weight management was offered	Significant reduction of AF burden and severity and benefits in cardiac remodeling
Upper airway stimulation					
Strollo Jr. et al. [53] (2014)		Prospective cohort study	126	UAS device was surgically implanted and objective and subjective measurements of severity of sleep apnea was done	Significantly improves the objective and subjective measurements of severity of the sleep apnea
Novel therapeutic modalities					
RDN	Linz et al. (2012) [54]	Animal study	20 anesthetized pigs	RDN was studied and anti-arrhythmic effect, sympathetic activity, post-apneic BP were studied	Reduced NTP induced AERP shortening inhibits post-apneic BP rise
LLVS	Gao et al. (2015) [55]	Animal study	11 rabbits	LLVS was studied in sleep apnea induced rabbits, and ERP, BP, intraesophageal pressure, blood gases measured before and after each episode of OSA	LLVS suppressed ERP shortening and AF induced by OSA
LL-RBS	Linz et al. (2016) [56]	Animal study	8 pigs	Pigs were induced sleep apnea for 3 hours. Changes in AERP and AF inducibility were verified	LL-BRS suppressed NTP induced AERP shortening and AF inducibility

Limitations

This review article has a few limitations. The article focuses solely on OSA's impact on AF for analysis and ignores other coexisting factors involved in the prognosis of the condition. The article covers minimal newer treatments as they require further research and evaluation. The sample size of most of the studies is minimal. Also, the paper has not explored the genetics common to the development of AF in OSA and its role in risk factor development.

## Conclusions

This review article addresses the association between OSA and AF and highlights previous research and studies. This article explores the mechanisms that lead to the development of OSA-associated AF and looks for appropriate management and treatment strategies. OSA has a negative impact on EC, ablation, and pharmacological therapies for AF. CPAP is considered the gold standard therapy for OSA and is known to reduce the risk of development and progression of AF. It has been beneficial in several aspects, including preventing arrhythmias irrespective of PVI or ablation or cardioversion, a significant reversal in electrical remodeling in AF progression, and reduction of MSNA, blood pressure, and catecholamine excretion. Several novel treatment modalities like UAS, RDN, LLVS, and LL-RBS have been tested for OSA-associated AF but warrant further research and developmental studies. This article’s clinical importance is to raise awareness among physicians, understand the mechanisms and risk factors involved, and attempt to reduce the development of AF in patients with OSA. Since the association is often not diagnosed, strict screening of these conditions can pave the way to improvement in the quality of life of patients. However, the information and studies on this topic are limited, and more comprehensive studies must be undertaken. In addition, the preventive strategies mentioned throughout the article need further evaluation and research in the future in order to be confirmed.
